# Unexpected Intraoperative Discovery of Gallbladder Volvulus in an Elderly Female: A Case Report

**DOI:** 10.7759/cureus.82696

**Published:** 2025-04-21

**Authors:** Isabella Moncada, Alyssa McMandon, Chad Byrd, Saptarshi Biswas

**Affiliations:** 1 Surgery, Grand Strand Medical Center, Myrtle Beach, USA; 2 Medicine, Edward Via College of Osteopathic Medicine, Spartanburg, USA

**Keywords:** acute calculous cholecystitis, biliary pathologies, gallbladder volvulus, necrosis, right upper quadrant abdominal pain

## Abstract

Gallbladder volvulus is a rare but potentially life-threatening condition that can closely mimic the clinical presentation of acute cholecystitis. It occurs when the gallbladder twists on its axis, potentially causing obstruction, ischemia, and necrosis. Early diagnosis and surgical intervention are crucial in preventing complications. An 81-year-old female presented to the emergency department with nausea, epigastric pain, and right upper quadrant (RUQ) abdominal pain. Physical examination revealed tenderness in the epigastric region and RUQ, a positive Murphy’s sign, and guarding in the RUQ. Laboratory testing demonstrated leukocytosis, and a computed tomography (CT) scan with intravenous (IV) contrast identified pericholecystic fluid, gallbladder wall thickening, surrounding inflammation, and a gallstone obstructing the gallbladder neck, leading to an initial diagnosis of acute calculous cholecystitis. Intraoperative findings revealed a gallbladder volvulus. A timely cholecystectomy was performed, and the patient recovered without significant postoperative complications. Gallbladder volvulus presents a diagnostic challenge due to its nonspecific symptoms, which can mimic those of biliary pathologies. Imaging modalities like ultrasound and CT have limited sensitivity, while magnetic resonance cholangiopancreatography or color Doppler sonography have shown promise in improving diagnosis. Early recognition and surgical intervention are essential to avoid severe outcomes. This case highlights the diagnostic challenges posed by gallbladder volvulus. Further research is needed to explore optimal diagnostic strategies for preventing poor clinical outcomes in patients with gallbladder volvulus.

## Introduction

Gallbladder volvulus is a rare and life-threatening condition that typically presents as an acute abdomen [1]. The estimated incidence is approximately 1 in 365,000 cases annually [2]. This condition predominantly impacts elderly females, with a median age of 77 years, and arises when the gallbladder twists along the axis of the cystic duct and artery [3]. The twisting may lead to ischemia, necrosis, and severe complications if not treated promptly [4]. Due to its nonspecific presentation and rare occurrence, gallbladder volvulus is most often discovered intraoperatively. We report the case of an 81-year-old female presenting with symptoms initially suggestive of acute calculous cholecystitis, with intraoperative findings revealing gallbladder volvulus. This case highlights the importance of considering gallbladder volvulus in the differential diagnosis of atypical presentations of biliary disease, particularly in high-risk populations.

This study was presented as a QuickShot at the Southeastern Surgical Congress Annual Meeting in February 2024.

## Case presentation

**Figure 1:**
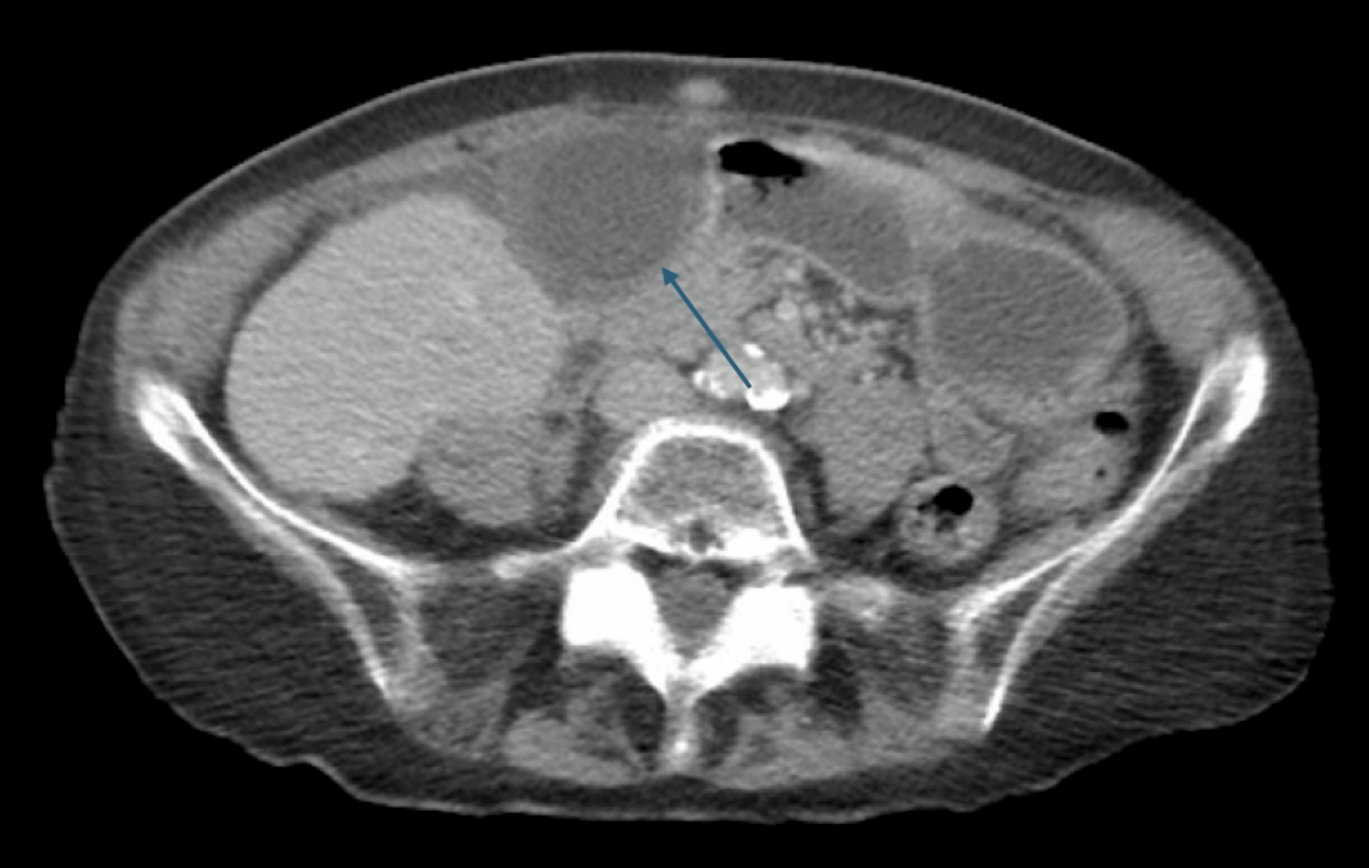
CT scan showing pericholecystic fluid, gallbladder wall thickening, and edema The blue arrow highlights the gallbladder with notable wall thickening and surrounding pericholecystic fluid and edema. CT: computed tomography

An 81-year-old female with a past medical history of hypertension and atrial fibrillation presented to the emergency department with sudden-onset epigastric and right upper quadrant (RUQ) abdominal pain, accompanied by nausea. Physical examination revealed epigastric tenderness, RUQ guarding, and a positive Murphy’s sign. Vital signs on admission were notable for elevated blood pressure, while all other parameters remained stable (Table 1). Laboratory testing demonstrated leukocytosis of 16.8 × 10³/μL, while other findings were unremarkable (Table 2). A computed tomography (CT) scan with intravenous (IV) contrast identified pericholecystic fluid, gallbladder wall thickening, surrounding inflammation, and a gallstone obstructing the gallbladder neck, leading to an initial diagnosis of acute calculous cholecystitis (Figure 1).

**Table 1:** Vital signs upon arrival to the emergency department BP: blood pressure, mmHg: millimeters of mercury, HR: heart rate, BPM: beats per minute, RR: respiratory rate, O2: oxygen

Variable	Value	Normal range
Temperature	97.3˚F	97-99˚F
BP	155/74	<120/80 mmHg
HR	84	60-100 bpm
RR	18	12-20 (breaths per minute)
Pulse oximetry	99%	95-100% O2

**Table 2:** Pertinent laboratory values upon arrival to the emergency department WBC: white blood cell count, AST: aspartate aminotransferase, ALT: alanine aminotransferase, ALP: alkaline phosphatase

Variable	Value	Unit	Normal range
WBC	16.8	x10³/µL	3.7-10.1
Hemoglobin	12.9	g/dL	11.6-15.4
Hematocrit	38.6	%	34.9-44.1
Platelets	240	x10³/µL	150-450
Total Bilirubin	1.1	mg/dL	0.1-1.1
AST	43	Units/L	15-46
ALT	49	Units/L	13-69
ALP	127	Units/L	38-126

**Figure 2:**
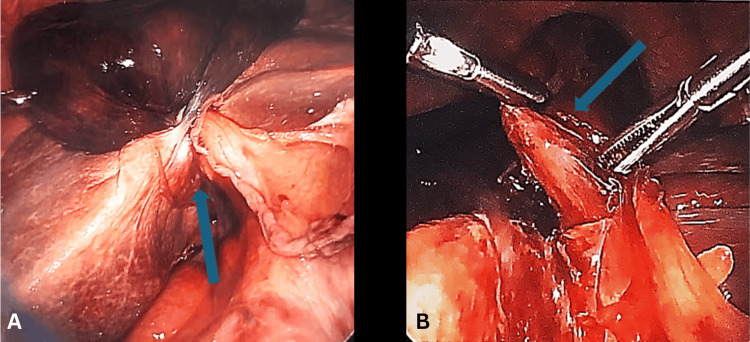
Intraoperative photo illustrating the gallbladder volvulus with ischemic changes before (A) and after (B) detorsion In Image A, the blue arrow indicates the gallbladder volvulus and the associated twisting of the mesentery. In Image B, the blue arrow illustrates the careful untwisting of the mesentery.

The patient underwent emergent laparoscopic cholecystectomy, where intraoperative findings revealed a gangrenous gallbladder volvulus at the infundibulum (Figure 2A), an uncommon and potentially life-threatening complication. The patient’s gallbladder was rotated clockwise to reestablish normal anatomical alignment (Figure 2B), and the cholecystectomy proceeded in standard fashion. On postoperative day one, the patient developed atrial fibrillation with rapid ventricular response, which was treated medically, and rate control was achieved. Otherwise, the patient’s postoperative course was uncomplicated, and they were discharged on postoperative day 4.

## Discussion

Gallbladder volvulus represents a surgical emergency that requires prompt intervention to prevent ischemic complications [1]. Its presentation, characterized by nonspecific symptoms, creates a significant diagnostic challenge. While this patient experienced an overall uncomplicated hospital course, gallbladder volvulus can potentially result in significant morbidity. The condition occurs when the gallbladder rotates within its mesentery and twists around the axis of the cystic duct and artery, resulting in vascular compromise, bile flow obstruction, and potential necrosis. Anatomical predispositions such as an elongated and wide mesentery or loss of visceral fat and elasticity due to aging can increase the risk of gallbladder volvulus [1]. Additional risk factors include advanced age (age >70), female sex, weight loss, and liver atrophy [5].

Gallbladder volvulus can be a complete or partial torsion. A complete torsion occurs with a rotation of the mesentery greater than 180°, commonly causing RUQ pain and vomiting. In contrast, an incomplete torsion (<180°) may cause intermittent pain comparable to biliary colic. The torsion can occur in a clockwise or counterclockwise direction [3].

Despite its severity, the preoperative diagnosis of gallbladder volvulus remains challenging due to its nonspecific clinical resemblance to common biliary pathologies [6]. A review by Nakao et al. of 245 gallbladder volvulus cases identified abdominal pain, nausea, vomiting, a palpable mass, and fever as common but nonspecific findings [7]. The patient’s exam findings of RUQ pain, nausea, and a positive Murphy’s sign exemplify the diagnostic overlap, with the volvulus only identified intraoperatively.

Imaging plays a pivotal role in diagnosing biliary disease and overcoming these diagnostic challenges, but the limitations of conventional modalities complicate preoperative identification. Ultrasound is the preferred first-line imaging modality for evaluating gallbladder pathologies due to its high sensitivity and specificity for detecting gallstones, biliary duct dilation, and inflammation [8]. Although ultrasound is the first line for other biliary pathologies, its sensitivity for detecting gallbladder volvulus is low. A potential finding on ultrasound is the “cystic duct knot sign,” a hyperechoic nodular appearance indicating potential torsion of the mesentery [9]. CT imaging can offer broader diagnostic utility for a patient’s RUQ pain but is also limited in diagnosing gallbladder volvulus [8,10]. A “whirl sign” can be seen on CT imaging, representing a twisted cystic artery and medial deviation of the extrahepatic common duct. However, it is a nonspecific finding for gallbladder volvulus [11]. Advanced imaging techniques, such as magnetic resonance cholangiopancreatography (MRCP), have shown promise in improving preoperative diagnosis. Usui et al. highlighted the first case of MRCP aiding in diagnosis. MRCP demonstrated characteristic findings, including a V-shaped distortion of the extrahepatic ducts, tapering and twisting interruption of the cystic duct, and a distended gallbladder deviated to the midline [12]. Additionally, MRCP will show high signal intensity on T1 imaging due to hemorrhage or necrosis [11]. These findings support that MRCP could help increase the preoperative diagnosis of gallbladder volvulus. Additionally, color Doppler sonography may be another modality to identify reduced blood flow in a torsed gallbladder, differentiating it from acute cholecystitis [7].

Surgical intervention remains the definitive treatment for gallbladder volvulus, with detorsion and cholecystectomy being the procedure of choice [2]. Cholecystectomy allows for decompression, detorsion, and removal of the affected organ, thereby helping to prevent complications such as ischemia, necrosis, or perforation [7,13]. In this case, timely surgical intervention enabled a successful resolution without complications, emphasizing the importance of prompt recognition and management. Hemodynamically unstable patients should not undergo surgical intervention due to the high-risk nature; instead, a percutaneous cholecystostomy should be performed, but cholecystectomy is the treatment of choice [14].

Increased awareness of gallbladder volvulus among clinicians, particularly those managing elderly patients with risk factors and atypical biliary symptoms, is essential for improving diagnostic accuracy and outcomes. Enhanced imaging protocols and broader access to MRCP or color. Doppler sonography may aid in this diagnosis, reducing delays in treatment and associated morbidity. Further studies on diagnosis and management could improve outcomes for patients with this rare but life-threatening condition.

## Conclusions

Gallbladder volvulus is a rare diagnosis that often mimics more common biliary conditions, contributing to delays in recognition and management. This case report highlights an uncommon intraoperative diagnosis of a volvulized, necrotic gallbladder in an elderly female initially presumed to have acute cholecystitis. The anatomical distortion and necrotic appearance of the gallbladder underscore the importance of maintaining diagnostic flexibility in elderly patients with nonspecific presentations. By illustrating the intraoperative findings and clinical decision-making, this case highlights the value of increased clinical suspicion and the potential role of advanced imaging in improving preoperative detection. Ultimately, it reinforces the need for timely surgical exploration when concern persists to prevent ischemic complications and ensure optimal outcomes.
